# Increased Leaf Bacterial Network Complexity along the Native Plant Diversity Gradient Facilitates Plant Invasion?

**DOI:** 10.3390/plants12061406

**Published:** 2023-03-22

**Authors:** Xiang-Deng Du, Jiang Wang, Congcong Shen, Jichen Wang, Zhongwang Jing, Li-Nan Huang, Zhen-Hao Luo, Yuan Ge

**Affiliations:** 1 State Key Laboratory of Urban and Regional Ecology, Research Center for Eco-Environmental Sciences, Chinese Academy of Sciences, Beijing 100085, China; 2 University of Chinese Academy of Sciences, Beijing 100049, China; 3 School of Life Science, Taizhou University, Taizhou 318000, China; 4 School of Life Sciences, Sun Yat-Sen University, Guangzhou 510275, China

**Keywords:** plant invasion, facilitation, leaf bacteria, network complexity, species richness, *Solidago canadensis* L.

## Abstract

Understanding the mechanisms of biological invasion is critical to biodiversity protection. Previous studies have produced inconsistent relationships between native species richness and invasibility, referred to as the invasion paradox. Although facilitative interactions among species have been proposed to explain the non-negative diversity–invasibility relationship, little is known about the facilitation of plant-associated microbes in invasions. We established a two-year field biodiversity experiment with a native plant species richness gradient (1, 2, 4, or 8 species) and analyzed the effects of community structure and network complexity of leaf bacteria on invasion success. Our results indicated a positive relationship between invasibility and network complexity of leaf bacteria of the invader. Consistent with previous studies, we also found that native plant species richness increased the leaf bacterial diversity and network complexity. Moreover, the results of the leaf bacteria community assembly of the invader suggested that the complex bacteria community resulted from higher native diversity rather than higher invader biomass. We concluded that increased leaf bacterial network complexity along the native plant diversity gradient likely facilitated plant invasion. Our findings provided evidence of a potential mechanism by which microbes may affect the plant community invasibility, hopefully helping to explain the non-negative relationship between native diversity and invasibility.

## 1. Introduction

The invasion of exotic species has become a global ecological issue, with potentially grave consequences for local biodiversity and ecosystem stability [1]. The identification of mechanisms that determine invasion success is a central question in ecology, especially in the context of biodiversity loss [2]. Many studies have found that a diverse community is more difficult to invade [3,4,5,6], which has largely been explained by complementarity and sampling effects. A greater richness of native species corresponds to a broader utilization of available resources, which in turn indicates a reduction in niche space available for exotic plants [7,8,9]. A diverse community may also resist an exotic plant by including a more competitive species, which is the so-called sampling effect [3,10]. While previous studies have shown a negative relationship between native species richness and community invasibility [8,11,12], it should be noted that other studies have demonstrated neutral or even positive relationships at both fine (<100 m^2^) and large scales (>1 km^2^) [1,13,14,15,16,17]. These inconsistent results indicate that other factors may modulate the invasion process in a covariant manner with species richness. The positive diversity–invasibility relationship at large scales is frequently contributed to habitat heterogeneity, whereby environmental factors, such as nutrient availability, may have comparable impacts on both native and exotic species [18,19]. However, the mechanism driving the non-negative diversity–invasibility relationship at fine scales is poorly understood.

Species interactions between native and exotic species are the key factor influencing invasion success [20]. Studies have found that interspecific facilitation between native and exotic species may play an important role in the invasion of exotic species [21,22,23]. Recent studies also noticed that soil microbes were crucial in the network of indirect effects on plant invasion [24,25,26]. Native plant diversity affects the invasion of non-native species through facilitative and competitive interactions, with the relative strength of facilitation and resistance regulating the net effect [27,28]. Native plants may support exotic plants directly by ameliorating local environmental conditions [29,30], or indirectly by decreasing herbivory or maintaining mutualists [31]. However, the pathways through which the facilitation of plant-associated microbes affect the invasion process remain largely unknown in the context of plant diversity and invasibility [23,32].

Plant diversity has been shown to positively correlate with plant-associated microbial diversity, network complexity and stability [33,34,35]. Moreover, more complex microbial networks could provide benefits to plants with a wider range of environmental fluctuations and suppress pathogen infection in plants [35,36,37]. Hence, plant-associated microorganisms may mediate the effects of plant richness on the invasiveness of exotic species, which is an overlooked process entangled in the diversity–invasibility relationship. As a part of the plant’s secondary genome [38], leaf microbes (both endo- and ecto-symbionts) play vital roles in plant growth and health, e.g., modifying plant hormone production [39], mitigating host resistance to abiotic and biotic stresses [40,41] and fixing atmospheric nitrogen [42]. However, most previous studies on plant microbiomes focused on soil microbiomes, overlooking leaf microbiomes. Compared with soil microbiomes, leaf microbiomes are reputed to be simpler [43]; it can be a more convenient factor with which to distinguish between native plants and exotic plants. Consequently, we postulated that the network complexity of leaf bacteria could be a covariate factor influencing the invasion resistance of plant species richness. We hypothesized that: (1) increasing native plant species richness would increase the diversity and network complexity of leaf bacteria on exotic species, and (2) diverse and complex leaf bacteria communities would presumably facilitate invasion. If true, our study’s results could elucidate a novel mechanism facilitating plant invasion.

To test these two hypotheses, we measured the leaf bacterial diversity and calculated the network complexity of the invader in a two-year field experiment (Tables S1 and S2). *Solidago canadensis* L. (Canada goldenrod) was chosen as the invader. It is a species that originated in North America and has successfully invaded central and western Europe, most of Asia, Australasia, and other regions [44,45]. A previous study showed that community stability significantly declined when heavily invaded by *S. canadensis* [46]. We used the biomass of *S. canadensis* to reflect the community invasibility, and a higher invader biomass to indicate higher community invasibility [47].

## 2. Results

### 2.1. Plant Richness and Invader Biomass

**Figure 1:**
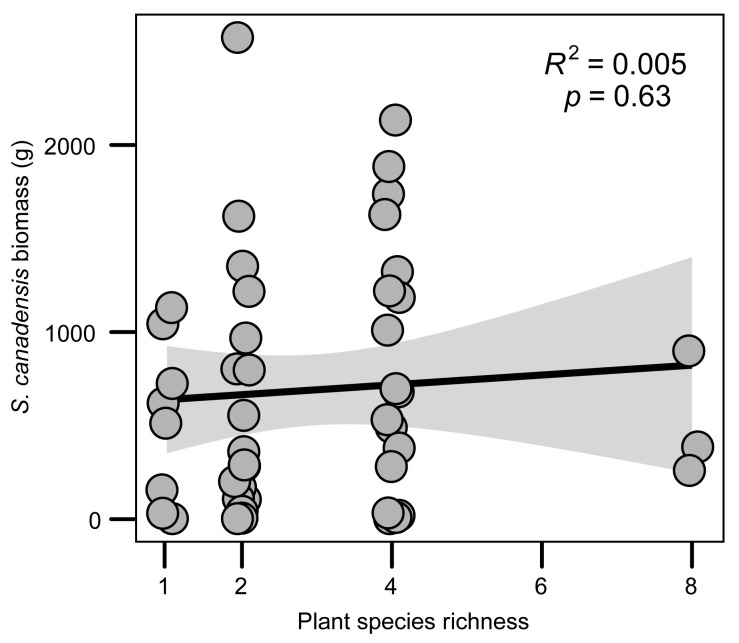
The relationship between invader biomass and native plant species richness.

The biomass of the invader did not decrease with native plant species richness (*R*^2^ = 0.005, *p* = 0.63, Figure 1). Considering that there were three replicates at the level of native plant species richness of 8, we checked the statistical significance without this plant species richness level. This did not significantly increase with native plant species richness (*R*^2^ = 0.038, *p* = 0.21, Figure S2).

### 2.2. Plant Richness and Leaf Bacteria Community

**Figure 2:**
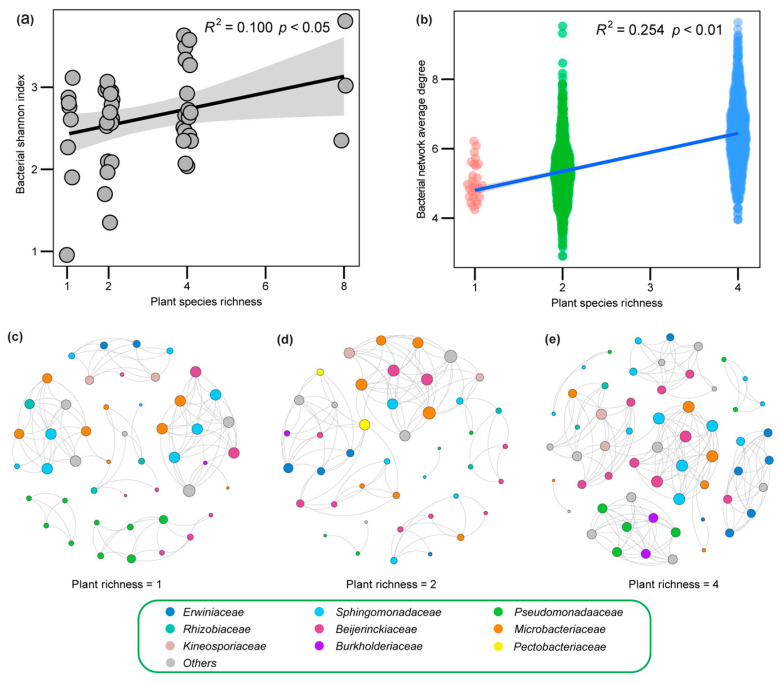
Alpha diversity and network complexity of leaf bacteria community of the invader along the native plant species richness gradient. (**a**) The relationship between bacterial alpha diversity and native plant species richness. (**b**) The relationship between bacterial network complexity and native plant species richness. (**c**–**e**) The selected presentative networks at three plant species richness levels. Node size is weighted by the number of its edges in each network. Nodes are colored based on their families. Others include unclassified taxa or those with low abundance in networks. As less than the minimum number of samples are used in constructing the network, the network complexity of the 8-species treatment is not calculated.

*Proteobacteria* and *Actinobacteria* are two dominant taxa groups at the phylum level (Figure S5). Shannon diversity (*R*^2^ = 0.100, *p* < 0.05, Figure 2a) and network complexity (*R*^2^ = 0.254, *p* < 0.001, Figure 2b) of leaf bacteria of the invader were positively related to native plant species richness. As the richness of native plant species increased, the relative abundance of *Beijerinckiaceae* also increased, while the relative abundance of *Erwiniaceae* decreased (Figure S6). Furthermore, the representative networks showed that native plant species richness complicated the bacterial network on the leaf of the invader (Average degree: 4.98, Figure 2c; 5.30, Figure 2d; 6.41, Figure 2e). *Beijerinckiaceae* occurred in most modules of three networks (Figure 2c–e).

### 2.3. Leaf Bacteria Community and Invader Biomass

**Figure 3:**
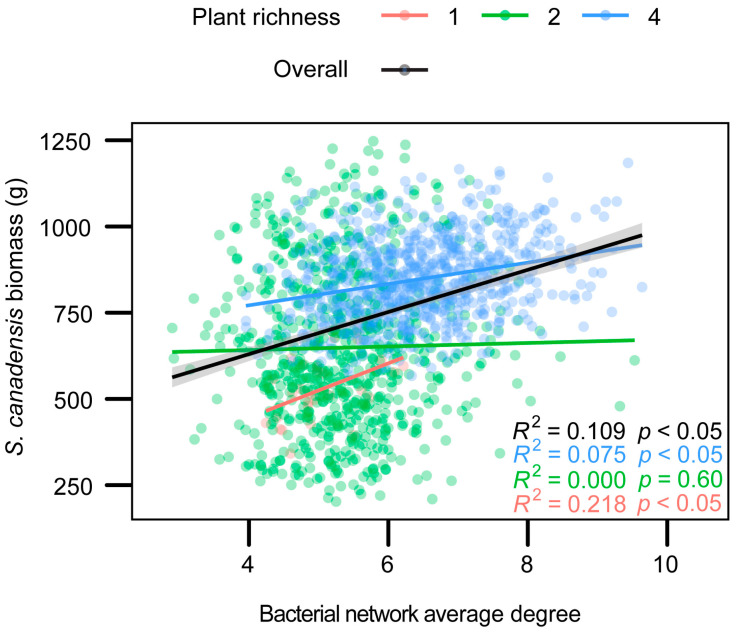
The relationship between invader biomass and bacterial network complexity. Points and lines represent different native species richness levels in red, green, and blue. The black line shows the overall trend of all data points, with shaded areas in the 95% confidence intervals.

Invader biomass did not significantly increase with leaf bacterial alpha diversity (*R*^2^ = 0, *p* = 0.99, Figure S3). However, except for the 2-species level (*R*^2^ = 0, *p* = 0.60), within species richness treatments, invader biomass positively correlated with the network complexity of leaf bacteria (1-species treatment *R*^2^ = 218, *p* < 0.05, 4-species treatment *R*^2^ = 0.075, *p* < 0.05, Figure 3). Moreover, across species richness treatments, invader biomass significantly increased with the network complexity of leaf bacteria (*R*^2^ = 0.109, *p* < 0.05, Figure 3).

### 2.4. Community Assembly of Leaf Bacteria of the Invader

**Figure 4:**
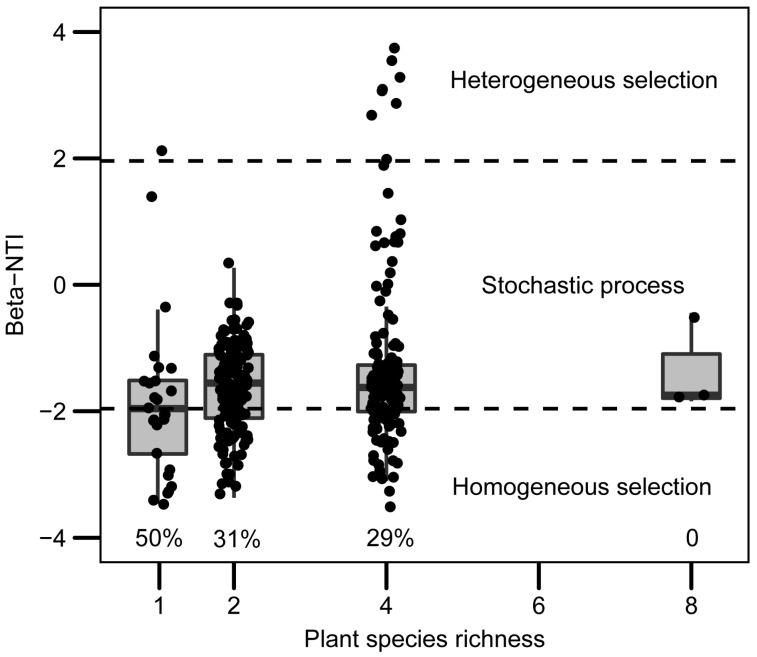
Distribution of beta-NTI values along native plant species richness gradient. Dash lines at beta-NTI = −1.96 and beta-NTI = 1.96 denote thresholds for the assembly process. The percentage below the box plot represents the proportion of homogenous selection.

Null model analysis showed that the relative contribution of deterministic (|βNTI| ≥ 1.96) and stochastic (|βNTI| < 1.96) processes of leaf bacteria assembly of the invader were significantly affected by native plant species richness (Figure 4). Higher native species richness resulted in a lower proportion of homogeneous selection (from 50% to 0%). Linear regression results indicated that the β-NTI was significantly correlated with native plant species richness (*R*^2^ = 0.026, *p* < 0.01, Figure S4).

## 3. Discussion

Niches-based theory predicts that species-rich communities will be more difficult for non-native plants to invade [48,49]. However, previous studies also found that diverse communities were actually invaded by more exotic plants than those communities including fewer species [13,50]. In this study, based on a two-year field biodiversity experiment, we found that: (1) the invader biomass did not increase with native plant species richness (Figure 1); (2) the invader biomass was positively correlated with the leaf bacterial network complexity of the invader (Figure 3); (3) community assembly process of leaf bacteria of the invader (Figure 4) and regression analysis (Figure 2) showed that more complex leaf bacteria communities resulted from native plant diversity rather than higher invader biomass. Our results suggested that the non-negative relationship between native diversity and invasibility may be due to the facilitation of complex leaf bacteria communities in the treatment with high native plant species richness.

While experimental evidence has firmly established the negative diversity–invasibility relationship at fine scales, predicted by niche-based theory within [1,51], some experimental studies have detected positive diversity–invasibility relationships at the same scale [17,52]. According to a synthesis study on small-scale native–exotic richness relationships, more than 60% of studies found negative correlations, and the rest found no correlation or mixed correlations [53]. By analyzing the native–exotic species richness relationship for vascular plants at three spatial grains (0.25 m^2^, 1 m^2^, 5 m^2^ of sampling grain) in 103 wetlands across Illinois, Chen et al. found exotic species richness was positively correlated with native species richness at 5 m^2^ (*r* = 0.334, *p* < 0.01, *n* = 103) and 1 m^2^ (*r* = 0.133, *p* < 0.01, *n* = 515) grain, and negatively correlated at 0.25 m^2^ (*r* = −0.154, *p* < 0.01, *n* = 2060) grain [15]. Another study, by Stohlgren, at 1 m^2^ grain across the United States, found native–exotic richness relationships were significantly positive in nearly all states (most of *r* > 0.6, *p* < 0.05) [19]. Consistent with this study, we also found a non-negative diversity–invasibility relationship at 4 m^2^ grain (*R*^2^ = 0.005, *p* = 0.63, Figure 1). By contrast, the correlation between invasibility and native species richness was low in our study (Figure 1), probably because of the small extent (47 plots within the site) of our field experiment, compared to the studies by Chen and Stohlgren. There could be some different process behind the plot size or study extent in driving inconsistent diversity–invasibility relationships [54]. Environmental heterogeneity was proposed to explain why diverse communities were more frequently invaded by exotic species at large scales [55]. However, little is known regarding the factors driving the non-negative diversity–invasibility relationship at fine scales [56]. Several studies pointed out that facilitative interactions could affect how native diversity promotes or resists exotic plants [23,27], but the facilitation of plant-associated microbes, in the context of invasibility and native diversity, has yet to be assessed. Through a field experiment with a low resolution (4 m^2^ per plot), we addressed this question, showing a positive correlation between the invader biomass and the network complexity of the leaf bacteria community of the invader. Furthermore, according to the results of the null model, we argued that the higher network complexity of the leaf bacteria community resulted from higher native plant diversity.

The positive correlation, shown in Figure 3, did not automatically mean that the change in the leaf bacterial network complexity of the invader was the cause of the change in the invader biomass, which is the proxy of invasibility. Specifically, it could be attributable to three phenomena. First, complex leaf bacteria communities could benefit invader growth, which was our hypothesis. Second, bacteria on leaves could be strongly selected by the host plant [38] and affected by neighbor habitats (i.e., soil and other plants) [57]. Given the taxa–area relationship [58], higher invader biomass could shape complex leaf bacteria communities of the invader by providing a larger and more uniform habitat. Third, complex soil bacterial communities in diverse plant communities could simultaneously increase leaf bacterial network complexity [38] and invader biomass [36]. To disentangle this conundrum, we used null model analyses to determine the community assembly process of leaf bacteria. Since higher invader biomass was expected to strengthen the influence of homogeneous selection, the decreasing homogenous selection along the native plant diversity gradient (Figure 4) ruled out the suspicion that higher invader biomass shaped complex leaf microbiomes. Additionally, as two parts of plant-associated microbiomes, leaf microbiomes should play a role similar to soil microbiomes in benefitting plants [59]. Complex leaf microbiomes should also benefit the invader if soil microbiomes do. Therefore, although we did not examine the role of soil bacteria, it was deemed reasonable to doubt that complex leaf bacteria communities of the invader could, to some extent, account for the greater invader biomass in diverse plant communities.

Growing evidence has shown that local community or other non-native species could directly or indirectly promote invasion [23,60], especially in a stressful environment where facilitative interactions occur more frequently [61]. For instance, a previous study showed that microclimates modified by native cushion plants facilitated the establishment and performance of an exotic species [29]. Although the facilitation of microbiomes in plant invasion has yet to be adequately studied, previous studies suggested that complex soil microbial networks might benefit plant growth more than simple ones [36,62]. Additionally, a complex microbial network could constrain pathogen proliferation [62]. *Erwiniaceae* are a Gram-negative family, and are pathogenic to numerous plants [63]. In this study, we found that the relative abundance of *Erwiniaceae* decreased as native plant richness increased (Figure S6), which could account for the constraint of networks with greater complexity at higher levels of plant diversity (Figure 2c–e). Herein, an invader associated with a complex leaf bacterial network was expected to thrive. Therefore, the facilitation of more complex leaf bacteria communities could neutralize the resistance of the local community to the exotic plant, even resulting in a positive diversity–invasibility relationship.

Leaf bacteria of *S. canadensis* were predominated by two phyla (*Proteobacteria* and *Actinobacteria*, Figure S5), which was consistent with previous studies on tomato leaves and other plant tissues. This could perhaps have been due to their ability to establish interactions with plants [64,65,66]. Remarkably, we did not find any evidence that invasibility was correlated with the alpha diversity of the invader’s leaf bacterial community (*p* > 0.05, Figure S3). Based on the assumption that a diverse microbial community would foster more beneficial microbes, some studies found that plant biomass positively correlated with root-associated microbial diversity [67]. However, a diverse bacterial community may also contain more harmful microbes, offsetting the promotion effect of beneficial microbes on plant biomass. Furthermore, there may be a trade-off between diversity and functionality when considering some specific functions [68]. Microbial diversity is not inherently good or bad [69], and does not necessarily denote a positive effect on invasibility. However, we found that, along the native plant diversity gradient, the relative abundance of *Beijerinckiaceae* increased (Figure S6). Previous studies identified the role of *Beijerinckiaceae* in N_2_ fixation [70]. We also found higher numbers of interactions of *Beijerinckiaceae* in the network at higher levels of plant diversity (Figure 2c–e). Together, these results implied that *Beijerinckiaceae* could be advantageous for invader growth. Further study on invasion should pay more attention to specific functional mechanisms of microbes.

Although we did not check the leaf bacteria communities on native plants, the lower relative contribution of homogeneous selection in diverse communities (Figure 4) implied that the complex leaf bacteria community resulted from higher native diversity, not from higher exotic plant biomass. Together with the positive correlation between the invader biomass and leaf bacterial network complexity, complex leaf bacteria communities of the invader presumably account for higher invader biomass. Notably, native plant species richness may also increase the diversity and complexity of leaf bacteria on native plant leaves, which benefits native species. However, we still found the invasibility dramatically increased with the network complexity of leaf bacteria, presumably because the exotic plant underwent environmental change upon comparing their original environment with that after introduction. The native plant would not do so; thus, the role of a complex bacterial community in coping with disturbances occurs only in the exotic plant. Furthermore, across species richness treatments, invader biomass significantly increased with the network complexity of leaf bacteria (Black regression line, Figure 3), implying the prevalence of the positive correlation between invader biomass and the network complexity of the leaf bacteria community of the invader.

## 4. Materials and Methods

### 4.1. Experiment Design

Our controlled biodiversity experiment was located in Taizhou city, Zhejiang province of China (28°31′ N, 121°23′ E). This region has a subtropical monsoon climate. During the time of this study, the mean annual temperature at the study site was 19 °C, and the mean annual precipitation was 1300 mm. The study site was an abandoned agricultural field, where the soil was classified as fluvo-aquic. After being abandoned in 2010, about 10 cm of topsoil was stripped in October 2016 to largely remove the seed bank. The site was weeded four times to further remove any remaining plants before transplanting seedlings. The native species pool consisted of 8 species (Table S1), which naturally grew in brush and grassland communities around Taizhou city. To create simulations of natural communities with distinct spatial and temporal niches, we selected species from four functional groups based on their height and growing season. These groups included tall species with an early growing season, short species with an early growing season, and so on. Additionally, seed availability was also considered in species selection since seeds were collected in the field. Given the two-year experimental duration, all these species were perennial or biennial. Our grassland experiment included 47 individual plots (each 2 × 2 m) randomly distributed at 1 m intervals (Figure S1). The 47 plots were randomly assigned to be seeded with 1, 2, 4, and 8 native species, respectively, using different species compositions (Table S2). All of the 8 species were grown in monocultures; 2- and 4-species treatments included 6 species compositions with 3 replicates (replicates meaning the same species composition); 8-species treatment included 1 species composition with 3 replicates.

Seeds for this experiment were collected in the nearby hills from September to October 2016 and sown in an area beside the plots in November 2016. Seedlings were transplanted into the 47 plots when their height reached about 5 cm in May 2017. Each plot received 32 individual native plants in total (each species with the same number of individuals), and seedlings of the same species were spaced using other species’ seedlings. Seedlings of native species were transplanted into the plots in May 2017, followed by transplanting the seedlings of *S. canadensis* a month later. Twelve individual seedlings of *S. canadensis* were uniformly distributed in the center area of 1.5 × 1.5 m. This experiment basically relied on natural precipitation. During the dry season, artificial watering was directly applied once a day to the soil surface to keep the soil wet until rainfall could accomplish this for us.

### 4.2. Sample Collection and Plant Biomass

On a sunny day in July 2018, 45 leaves (15 leaves each from the upper, middle, and lower canopy) were randomly harvested from 5 individuals of *S. canadensis* in each plot. Leaves were picked wearing nitrile gloves, sealed in plastic bags, and immediately stored in an icebox. In October 2018, the above-ground biomass of each species was harvested, then dried at 80 °C for 48 h and weighed.

### 4.3. Laboratory Analyses

Microbial DNA was extracted from leaf samples using the FastDNA Spin Kit for Soil (MP Biomedicals, Santa Ana, CA, USA) according to the manufacturer’s protocols. The final DNA concentrations and purification were determined by a NanoDrop 2000 UV-vis spectrophotometer (Thermo Scientific, Wilmington, NC, USA), and DNA quality was checked by 1% agarose gel electrophoresis. The V6–V7 hypervariable regions of the bacteria 16S rRNA gene were amplified with primers 799F (5′-AACMGGATTAGATACCCKG-3′) and 1193R (5′-ACGTCATCCCCACCTTCC-3′) [71] by thermocycler PCR system (GeneAmp 9700, ABI, Waltham, MA, USA). The PCR reactions were conducted using the following program: 3 min of denaturation at 95 °C, 13 cycles of 30 s at 95 °C, 30 s for annealing at 55 °C, and 45 s for elongation at 72 °C, with a final extension at 72 °C for 10 min. PCR reactions were performed in triplicate using a 20 μL mixture containing 4 μL of 5 × FastPfu buffer (TransGen AP221-02: TransStart Fastpfu DNA Polymerase; Shanghai Majorbio Bio-Pharm Technology Co., Ltd., Shanghai, China), 2 μL of 2.5 mM dNTPs, 0.8 μL of each primer (5 μM), 0.4 μL of FastPfu polymerase and 10 ng of template DNA. The PCR products were extracted from a 2% agarose gel and further purified using the AxyPrep DNA Gel Extraction Kit (Axygen Biosciences, Union City, CA, USA), then quantified using QuantiFluor™-ST (Promega, Madison, WI, USA), according to the manufacturer’s protocol. NEXTFLEX Rapid DNA-Seq Kit was used to construct Illumina sequencing libraries.

### 4.4. Sequence Processing

Samples were sequenced on the Illumina MiSeq platform. Raw paired-end fastq files were demultiplexed, quality-filtered by Trimmomatic, and merged by FLASH with the following criteria: (i) the reads were truncated at any site receiving an average quality score <20 over a 50 bp sliding window; (ii) primers were exactly matched, allowing 2 nucleotide mismatching, and reads containing ambiguous bases were removed; (iii) sequences whose overlap was longer than 10 bp were merged according to their overlap sequence. Based on trimmed sequences with an average length of 395 bp, we used the R package DADA2 to correct amplicon errors and to identify chimeras [72]. The end product of DADA2 yielded a total of 1666 unique amplicon sequence variants (ASVs). After being rarefied, we obtained 1626 unique ASVs. These were assigned taxonomic identifiers using the Silva reference database clustered at 99% similarity [73].

### 4.5. Statistical Analyses

In our study, community invasibility was assessed by the biomass of *S. canadensis*, i.e., a higher biomass of *S. canadensis* suggested a stronger invasion of the local plant community. Leaf bacterial alpha diversity was estimated by its Shannon diversity using the vegan package in R v4.1.2 [74]. The average degree (the average number of edges per node) was calculated as a proxy for the complexity of the bacterial network, which reflected the extent of interactions between bacterial species. Networks were constructed from Pearson correlations using the igraph package in R, in which correlations were considered when the absolute value of Pearson’s *r* > 0.8 and *p* < 0.05. Considering the different replicates at different plant species richness levels and the minimum number of samples to construct the bacterial network, we used 6 samples, each time, at each plant species richness level. For the single-species treatment, all sample combinations (6 samples per combination) from 8 samples were selected, including 28 unique networks. For 2-species and 4-species treatments, each sample was chosen from each plant combination (6 plant combinations in total), including 729 unique networks. Since it was less than the minimum number of samples constructing the network, the network complexity of the 8-species treatment was not calculated. Then, we manually selected the closest representative network to the average level at the same plant species richness level. All correlations were analyzed by linear regression.

To ascertain the deterministic or stochastic processes and their relative contribution to the community assembly, null model analysis was performed (1000 randomizations) using the iCAMP package in R. According to the framework based on the beta nearest-taxon-index (beta-NTI) [75,76], beta-NTI value < −1.96 indicated that homogeneous selection governed the community assembly process, whereas a beta-NTI > 1.96 indicated a dominant heterogeneous selection. A beta-NTI between −1.96 and 1.96 indicated a dominant stochastic process.

## 5. Conclusions

Our results found that, along the native plant diversity gradient, increased leaf bacteria network complexity of the exotic plant was positively correlated with the invasibility of the plant community. Furthermore, results of bacteria community assembly indicated that a more complex leaf bacteria community resulted from higher native plant diversity, rather than higher invader biomass. Therefore, through a diversity–invasibility experiment, we hypothesized that the leaf bacterial community presumably mediated the facilitation of local plant communities on exotic plants. This potential mechanism could reconcile the conflicting results of studies examining the relationships between native plant species richness and invasibility. To gain a more comprehensive understanding of how leaf bacteria influence the diversity–invasibility correlation, forthcoming studies should pay more attention to plant functional groups, the role of complex microbial communities on plant growth, and factors influencing the plant–microbe diversity relationship.

## Data Availability

The raw sequence data can be obtained with the accession number PRJNA832704 in the National Center for Biotechnology Information (NCBI). The raw data and code used for analyses are available at https://github.com/YuanGe-Lab/Xiangdeng_Du/tree/main/leaf_bacteria (accessed on 26 May 2022).

## Supplementary Materials

The following supporting information can be downloaded at: https://www.mdpi.com/article/10.3390/plants12061406/s1, Figure S1: Photograph of the experiment site, including 47 individual plots (each 2 × 2 m); Figure S2: The relationship between the biomass of *S. canadensis* and native plant species richness without 8-species treatment; Figure S3: The relationship between biomass of *S. canadensis* and bacterial alpha diversity. The overall trend across native species treatments (a). Points and lines in different colors represent four native species richness levels (b); Figure S4: Beta-NTI values of the community assembly process of leaf bacteria; Figure S5: Relative abundance of leaf bacteria taxa of the invader at the phylum level. The numbers above bars show the native plant species richness; Figure S6: Relative abundance of the families of the top 40 most abundant ASVs; Table S1: Information of the species pool used in this study. No. 1 is alien species; Table S2: Species composition of 47 plant communities. Abbreviations of species names are described in Table S1. Numbers below the species show the quantity of each species in a plot (community). Comm. is the abbreviation of community.
