# Supplementary material for: Increased Leaf Bacterial Network Complexity along the Native Plant Diversity Gradient Facilitates Plant Invasion?
Source: Plants (Basel). 2023 Mar 22;12(6):1406. doi: 10.3390/plants12061406 (PMC10052042; doi:10.3390/plants12061406)
Supplement: Supplementary file 1 [file plants-12-01406-s001.zip › plants-2214498-supplementary.pdf]

Supplemental material

Figure S1. Photograph of the experiment site, including 47 individual plots (each 2m × 2m).

Figure S2. The relationship between the biomass of *S. canadensis* and native plant species richness without 8-species treatment.

Figure S3. The relationship between biomass of *S. canadensis* and bacterial α diversity. The overall trend across native species treatments (**a**). Points and lines in different colors represent four native species richness levels **(b)**.

Figure S4. Beta-NTI values of the community assembly process of leaf bacteria.

Figure S5. Relative abundance of leaf bacteria taxa of the invader at the phylum level. The numbers above bars show the native plant species richness.

Figure S6. Relative abundance of the families of the top 40 ASVs.

Table S1. Information of the species pool used in this study. No. 1 is alien species.

Table S2. Species composition of 47 plant communities. Abbreviation of species names are described as in Table S1. Numbers below the species shows the quantity of each species in a plot (community). Comm. is the abbreviation of community.

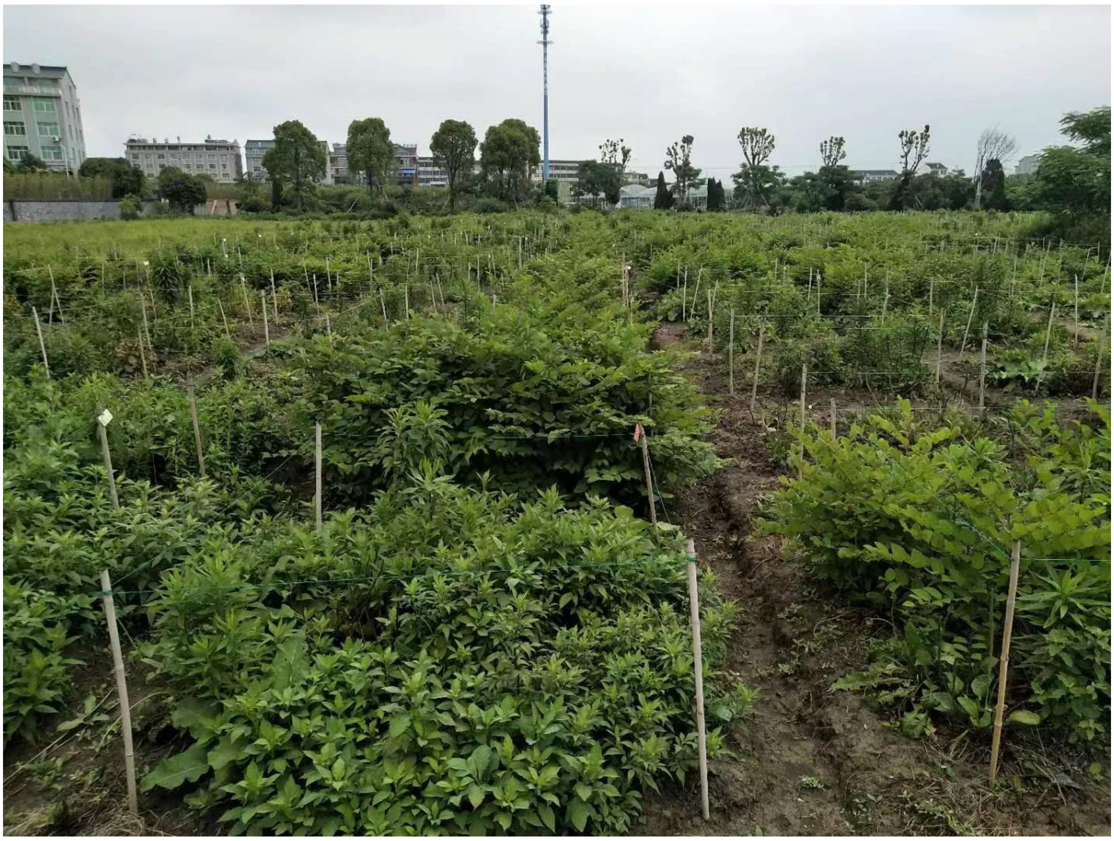

Figure S1. Photograph of the experiment site, including 47 individual plots (each 2m × 2m).

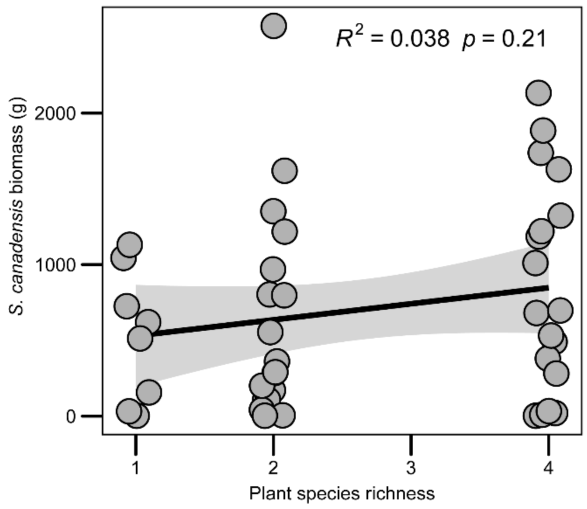

Figure S2. The relationship between the biomass of *S. canadensis* and native plant species richness without 8-species treatment.

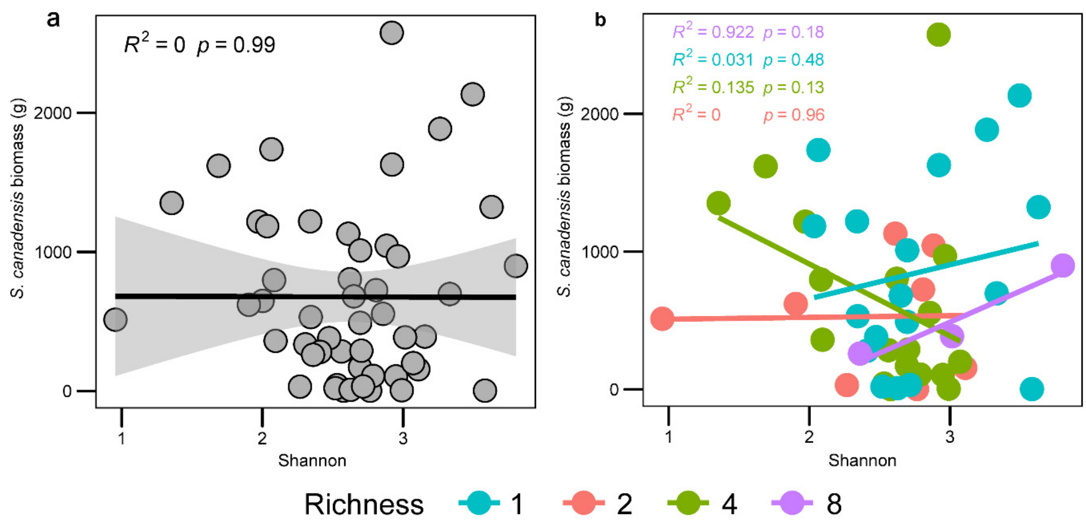

Figure S3. The relationship between biomass of *S. canadensis* and bacterial α diversity. The overall trend across native species treatments (**a**). Points and lines in different colors represent four native species richness levels **(b)**.

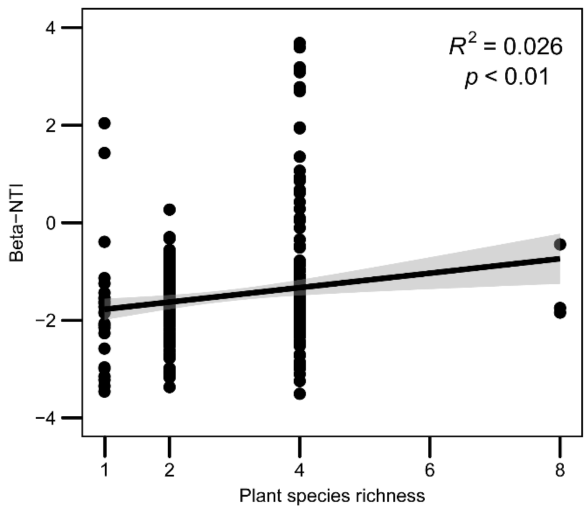

Figure S4. Beta-NTI values of the community assembly process of leaf bacteria.

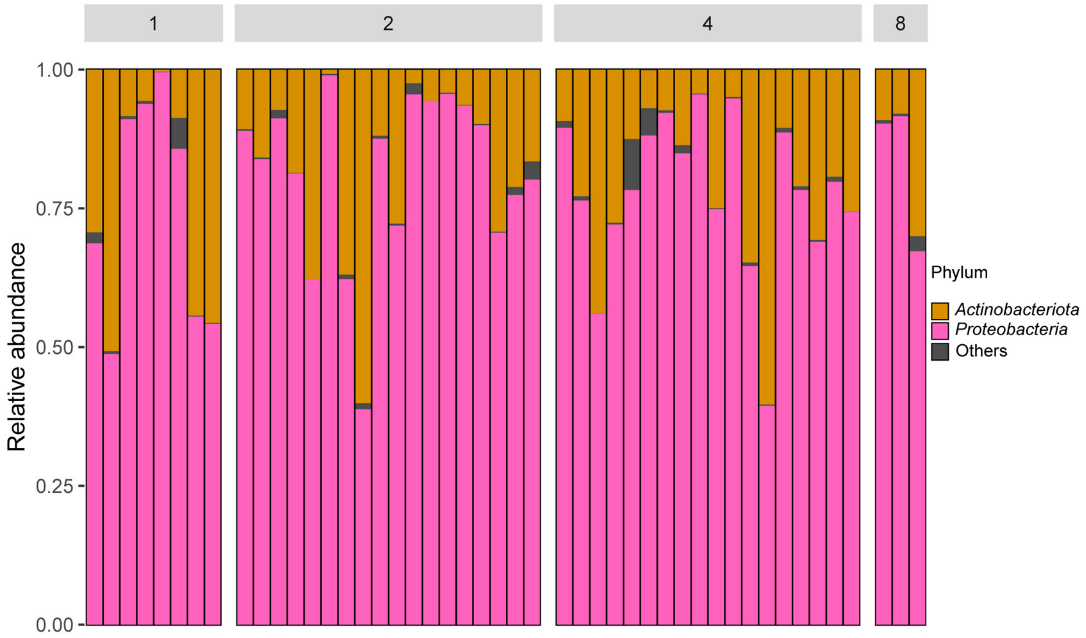

Figure S5. Relative abundance of leaf bacteria taxa of the invader at the phylum level. The numbers above bars show the native plant species richness.

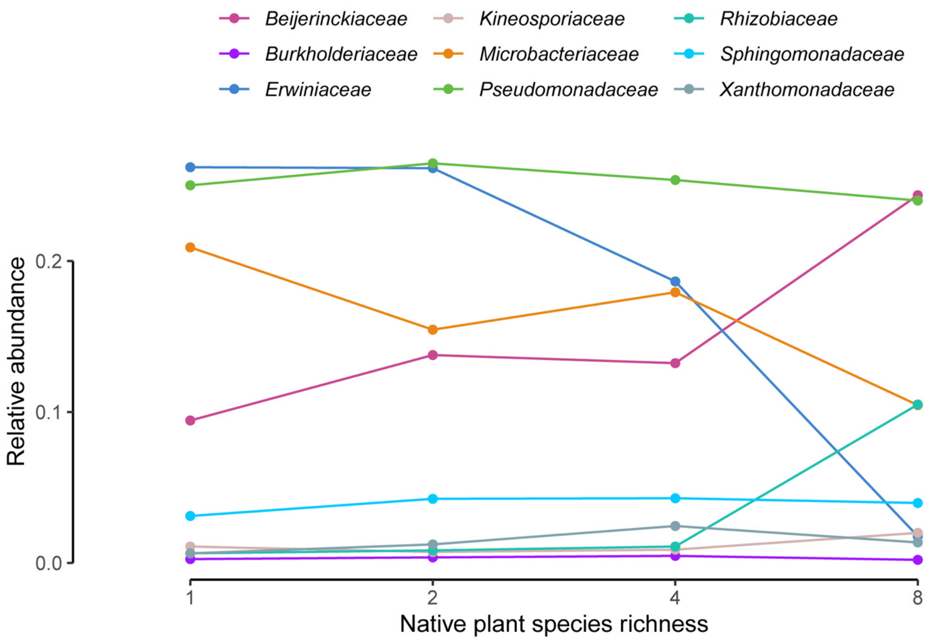

Figure S6. Relative abundance of the families of the top 40 most abundant ASVs.

Table S1. Information of the species pool used in this study. No. 1 is alien species.

No.	Species	Family	Life form	Functional trait	Abbreviation
1	*Solidago canadensis* L.	*Asteraceae*	Perennial	/	Sc
2	*Arctium lappa* L.	*Asteraceae*	Biennial	Tall; early growing season	Al
3	*Patrinia scabiosifolia* Fisch. ex Trev.	*Caprifoliaceae*	Perennial	Tall; early growing season	Ps
4	*Achyranthes bidentate* Blume	*Amaranthaceae*	Perennial	Tall; late growing season	Ab
5	*Reynoutria japonica* Houtt.	*Polygonaceae*	Perennial	Tall; late growing season	Fj
6	*Taraxacum officinale* F. H. Wigg.	*Asteraceae*	Perennial	Short; early growing season	To
7	*Plantago asiatica* L.	*Plantaginaceae*	Perennial	Short; early growing season	Pa
8	*Platycodon grandifloras* A. DC.	*Campanulaceae*	Perennial	Short; late growing season	Pg
9	*Antenoron filiforme* (Thunb.) Rob. et Vaut.	*Polygonaceae*	Perennial	Short; late growing season	Af

Table S2. Species composition of 47 plant communities. Abbreviation of species names are described as in Table S1. Numbers below the species shows the quantity of each species in a plot (community). Comm. is the abbreviation of community.

Comm. No.	Species richness	Invader	Native species							
		Sc	Al	Ps	Ab	Fj	To	Pa	Pg	Af
1	1	12	32							
2	1	12		32						
3	1	12			32					
4	1	12				32				
5	1	12					32			
6	1	12						32		
7	1	12							32	
8	1	12								32
9	2	12	16		16					
10	2	12	16		16					
11	2	12	16		16					
12	2	12	16				16			
13	2	12	16				16			
14	2	12	16				16			
15	2	12		16					16	
16	2	12		16					16	
17	2	12		16					16	
18	2	12			16		16			
19	2	12			16		16			
20	2	12			16		16			
21	2	12				16			16	
22	2	12				16			16	
23	2	12				16			16	
24	2	12					16			16

25	2	12					16			16
26	2	12					16			16
27	4	12	8	8	8	8				
28	4	12	8	8	8	8				
29	4	12	8	8	8	8				
30	4	12	8	8			8	8		
31	4	12	8	8			8	8		
32	4	12	8	8			8	8		
33	4	12	8	8					8	8
34	4	12	8	8					8	8
35	4	12	8	8					8	8
36	4	12			8	8	8	8		
37	4	12			8	8	8	8		
38	4	12			8	8	8	8		
39	4	12			8	8			8	8
40	4	12			8	8			8	8
41	4	12			8	8			8	8
42	4	12					8	8	8	8
43	4	12					8	8	8	8
44	4	12					8	8	8	8
45	8	12	4	4	4	4	4	4	4	4
46	8	12	4	4	4	4	4	4	4	4
47	8	12	4	4	4	4	4	4	4	4
